# Virtual screening of human pancreatic lipase inhibitors using an integrated transfer learning and molecular dynamics simulation

**DOI:** 10.6026/973206300223002

**Published:** 2026-06-30

**Authors:** C Tamilselvan, B.S Lakshmi

**Affiliations:** 1 Department of Biotechnology, Anna University, Guindy, Chennai, 600025, Tamilnadu, India

**Keywords:** Human pancreatic lipase (hPL), Transfer learning, Virtual screening, Prenylated flavanonol, Molecular dynamics simulation, Anti-obesity drug discovery

## Abstract

Human pancreatic lipase (hPL), a major enzyme involved in dietary lipid digestion is a crucial therapeutic target for the management of obesity. Therefore, it is of interest to identify hPL inhibitors using an integrated transfer learning and molecular dynamics simulation. Transfer learning predicted 17,309 active candidates based on the pIC_50_, from which prenylated flavanonol was identified as the top-ranked compound with a binding affinity of 9.85 kcal/mol in comparison to reference inhibitor orlistat using Glide XP docking. Moreover, 200ns molecular dynamics simulations confirmed the stability of the protein-ligand complex through sustained interactions with key catalytic residues, including Ser152 and Asp79. Thus, data shows that prenylated flavanonol as a promising natural inhibitor of hPL.

## Background:

Obesity is a significant worldwide health epidemic, leading to an increase in the risk of type 2 diabetes, cardiovascular diseases and some cancers. An excessive intake of dietary fats primarily triglycerides, are the major contributors of obesity, which are then hydrolyzed into monoglycerides and fatty acids in the intestinal lumen by lipases [1]. Human Pancreatic Lipase (hPL) is a critical digestive enzyme and is majorly responsible for hydrolysis of total lipids in the body. The hPL comprises of 449 amino acids with the catalytic triad of Asp176, Ser 152 and His 263. Modulation of the activity of human pancreatic lipase (hPL) is a promising therapeutic strategy for the inhibition of dietary fat absorption, thereby offering a novel approach for the treatment of obesity and its associated metabolic disorders [2]. Synthetic drugs like Phentermine, Orlistat, Contrave, Saxenda, Plenity, Imciveree, Wegovy and Tirzepatide are amongst the commonly prescribed drugs for the management of obesity and act by inhibition of pancreatic lipase. However, prolonged intake of these drugs has been reported to cause side effects including diarrhea, nausea, vomiting, constipation, Gut disorder, skin irritation and insomnia [3, 4]. Recent studies have shown natural products to exhibit good potential in the therapeutic management of obesity through improving metabolism, regulating blood sugar level, decreasing appetite, reducing impairment of key digestive enzymes, increasing insulin sensitivity, restricting development of new fat cells, and facilitating the breakdown of existing fat cells [5]. Natural compounds like 1,2,3,4,6-penta-O-galloyl-β-D-glucopyranose (PGG) and catechin gallate (CG) from Rhodiola crenulata have been identified as potent non-competitive hPL inhibitors, exhibiting very low inhibition constants (0.012μM and 0.082μM, respectively). These natural compounds are more advantageous over systemic treatments by minimizing adverse effects and maximizing therapeutic efficacy in the gastrointestinal tract [6].

Molecular docking analysis has shown amentoflavone to be highly potent against human pancreatic lipase providing a better alternative to orlistat. However, drug-likeness screening using Lipinskis rule of five favored naringenin 7-O-D-glucuronide as the best oral drug candidate [7]. Ibragimova *et al.* observed that the Drug Target interaction (DTI) prediction models based on molecular similarity, differed in their activities, causing a highly discontinuous Structure-Activity Relationships (SARs). To overcome this, Transfer Learning (TL) approach was utilized, wherein, a universal model Compound Encoder was first pretrained on a high-difficult, binary Activity Cliff (AC) prediction task (source domain) to learn robust AC-aware feature representations. This knowledge was then transferred and fine-tuned for a continuous value DTI prediction task (target domain), enhancing the DTI models generalization and robustness against AC phenomena [8]. Studies have shown a combined approach using molecular docking and molecular dynamics (MD) simulations to be highly effective in the design of new inhibitor drugs capable of overcoming Multi drug-Resistance (MDR-TB) and Extensively Drug-Resistant (XDR-TB) tuberculosis [9]. Therefore, it is of interest to describe virtual screening of human pancreatic lipase inhibitors using an integrated transfer learning and molecular dynamics.

## Materials and Methods:

## Data curation:

Data set from ChEMBL [10], BindingDB [11], NPASS database [12] and literature, comprising of chemical structures and bioactivity values of 332 compounds with their reported experimental IC_50_ values against human Pancreatic Lipase (hPL) was acquired. To ensure uniformity, a data set with similar units (nM) of IC_50_ values were retained during the curation process. For compounds with multiple reported activities, a mean value was calculated using the Pandas (v2.2.0) library in Python, and one entry was utilized. The processed data set finally consisted of 321 compounds. For further classification, compounds with reported IC_50_ values >=6 were labelled as active, whereas molecules with IC_50_ <6 were labelled as inactive. This resulted in 183 active compounds and 138 inactive compounds.

## Transfer learning:

The chemical compounds, were represented as SMILES strings, and converted into 167-bit MACCS fingerprints using the RDKit library for numerical representation. The pIC_50_ value, was preprocessed applying Standard Scaler for normalization. The dataset was then divided into training and validation sets in an 80:20 ratio, with a fixed random state applied to ensure reproducibility. The predictive model architecture was built using Tensor Flow Keras, comprising an input layer, two hidden layers with 128 and 64 neurons respectively (both utilizing the ReLU activation function), and a single-neuron output layer with a linear activation function. The model was trained for 100 epochs using the Adam optimizer to minimize the mean squared error (MSE), while the mean absolute error (MAE) served as a key performance metric. To reduce the risk of overfitting, an early stopping callback was implemented to monitor validation loss with a patience of 10 epochs, ensuring that the weights of the models were restored for optimal performance.

## Selection and preparation of protein:

The 3D X-ray diffraction structure of human pancreatic lipase (PDB ID: 1LPB) was retrieved from the RCSB Protein Data Bank. Prior to docking, protein structure was prepared using the Protein Preparation Wizard in Maestro (Schrödinger Suite), which involved assigning correct bond orders, adding missing hydrogen atoms, rebuilding of incomplete side chains and removing crystallographic water molecules. The prepared structure was then optimized and energy minimized using the OPLS force field to relieve steric clashes and obtain a stable conformation ensuring a stable and reliable conformation for subsequent docking calculations.

## Selection and preparation of ligands:

The structures of the selected ligands were obtained from the lotus Database. Maestro LigPrep was used to prepare the ligands for docking studies. The ligands structures were energetically optimized with a default setting of the LigPrep module, and the ionization state was set to provide each possible state at pH 7.0 ±2.0.

## Molecular docking:

Molecular docking was performed to predict the binding conformation and interaction of ligands with the protein using Glide module in Maestro. A receptor grid was generated around the active site of human pancreatic lipase. During grid generation, a Van der Waals scaling factor of 1.0 and a partial charge cutoff of 0.25e were applied. The Glide Extra Precision (XP) mode of Schrodinger, which provides enhanced accuracy in predicting ligand binding conformation was utilised. The prepared ligands were docked into the defined active site of human pancreatic lipase, and the resulting docking scores were analyzed to estimate their binding affinity and orientation within the binding pocket.

## Molecular Dynamics (MD) simulations:

Molecular dynamics (MD) simulations were carried out using the Desmond module of the Schrodinger software suite. The protein-ligand complex of the top-ranked analogue, identified based on docking score, was selected for simulation. The system was prepared using the System Builder panel, where the complex was placed in an orthorhombic simulation box and solvated with the Simple Point Charge (SPC) water model. The simulation was performed for 200 ns under an NPT ensemble at a temperature of 310.15 K and a pressure of 1.013 bar, representing physiological conditions. Periodic boundary conditions were applied to maintain a realistic environment. Post-simulation, the Simulation Interaction Diagram tool was employed to analyze the trajectories and evaluate protein-ligand interactions over time. All simulation parameters and analyses were conducted using the built-in features of Desmond.

## Results and Discussion:

## Transfer learning analysis:

## Pretraining phase:

A pre training model was initially developed and trained on a large dataset (Hydrolase Dataset) from ChEMBL_35 containing pIC_50_ values to acquire foundational knowledge about structure-activity relationships of unique hydrolase compounds (16,174). A feedforward neural network (FFN) was utilised with an architecture designed to predict continuous bioactivity (pIC_50_) values. The model was optimized using Adam optimizer with a mean squared error (MSE) loss function, and an early stopping strategy to prevent overfitting. Results show both the training and validation curves consistently declined downward and then stabilized, indicating that the model successfully converged without significant overfitting. The model's performance on the validation set resulted in an MAE of 0.680 and a R^2^ of 0.539, indicating the efficiency of the pretrained model (Figure 1 - see PDF). This increasing divergence between the losses suggests the model continuously improved its fit to the specific training data but failed to generalize these gains to the validation set (Figure 1 - see PDF).

## Fine-tuning phase:

To adapt the learned knowledge of the compounds to a more specific task of identification of inhibitor compounds, the pretrained model was fine-tuned with a smaller dataset, the PL dataset containing 321 compounds. The learning curves for the fine-tuned model are shown in (Figure 2 - see PDF) displaying the MAE and Loss over epochs. Results show that the model converged very quickly and did not diverge, confirming that it wasn't overfitting the new data. This strong predictive performance demonstrates that the pre-trained model gained the required knowledge allowing it to generalize well to the new dataset. The fine-tuned model achieved an impressive MAE of 0.165 and a R^2^ of 0.911 on the validation set, which confirms its high accuracy and reliability in predicting pIC_50_ values. Out of the total predicted compounds of 1, 09,669 using the fine-tuned model 17,309 compounds were classified as active pIC_50_ >= 6.0), while the remaining 92,360 compounds were labeled as inactive. This classification provided a refined subset of potentially bioactive candidates suitable for further prioritization.

## Cross-dataset Validation of Model Generalizability:

To confirm that the model could accurately predict the bioactivity of unknown compounds, the Kernel Density Estimation (KDE) was performed. Figure 3 (see PDF) shows the PNLIP fine-tuned dataset and the predicted lotus dataset distributions to exhibit similar shapes with overlapping peaks in the biologically relevant range of pIC_50_ between 6-8, indicating that the model successfully transferred its predictive capabilities. While the Predicted lotus dataset had a slightly broader distribution, reflecting greater chemical diversity, the close alignment of the curves prove the model's robustness and ability to make well-calibrated predictions across different chemical structures.

## Molecular docking analysis:

The active compounds 17,309 in the LOTUS database were then screened using the transfer learning model and further assessed using molecular docking analysis to determine their binding affinity to human pancreatic lipase (PDB ID: 1LPB). The Binding energy and interaction details of the top four docked compounds with 1LPB are tabulated in Table 1. Prenylated Flavanonol showed the highest extra precision (XP) docking score of -9.85 kcal/mol, which suggests a strong response to the active site of pancreatic lipase. Comparatively, Orlistat, the pancreatic lipase inhibitor used as positive control, exhibited a docking score of -3.81 kcal/mol under similar settings. The significantly lower docking score observed for Prenylated Flavanonol suggests a stronger predicted binding affinity toward the enzyme compared to orlistat, highlighting its potential as a promising inhibitor of pancreatic lipase. Analysis of the 2D docking interaction profiles showed the Lotus-derived compounds to exhibit similar interaction pattern as reference inhibitor orlistat (Figure 4 - see PDF). A majority of the compounds were observed to interact with key residues which are involved in binding of the substrate and catalysis like Ser152, Phe77, Leu153, Ala259, and Leu264. Prenylated flavanonol and LTS0199947 exhibited a relatively stronger interaction network due to the presence of multiple hydroxyl groups that are able to establish hydrogen bonds with the catalytic residues.

## ADME property analysis:

To assess the physicochemical profile of the predicted compounds, the SwissADME bioavailability radar plot analysis was performed with reference inhibitor Orlistat as the positive control. The radar plot represents important descriptors such as molecular weight, lipophilicity (LogP), polarity (TPSA), hydrogen bonding capacity, and molecular flexibility. Orlistat was observed to be highly hydrophobic and flexible with a lipophilicity of 7.05 (Consensus LogP), Molecular weight of 495.73 grams/mol, TPSA of 81.7 A^2^, 5 hydrogen bond acceptors, a single hydrogen bond donor and 24 rotatable bonds (Figure 5 - see PDF). These properties align with their mechanism of action as a lipase inhibitor primarily in the digestive tract. The docked lead compounds Prenylated Flavanonol (LTS0196517), LTS0218789, LTS0199947 and LTS0212803 exhibited varied physicochemical characteristics. Prenylated Flavanonol (LTS0196517) with a molecular weight of 456.49 g/mol, showed a TPSA of 147.68 A^2^ and a Consensus LogP of 2.78 with 8 hydrogen bond acceptors, 6 hydrogen bond donors, and 7 rotatable bonds, with moderate polarity and balanced lipophilicity. LTS0218789, with a molecular weight of 454.56 g/mol, showed a TPSA of 89.90 A^2^, a Consensus LogP of 4.11 with 6 hydrogen bond acceptors, 1 hydrogen bond donor, and 7 rotatable bonds, indicating good membrane permeability and drug-like properties. The LTS0199947 with a much higher molecular weight (864.75 g/mol) showed a TPSA (340.10 A^2^) a Consensus LogP of 1.00, with 20 hydrogen bond acceptors and 12 hydrogen bond donors, indicated a highly polar structure that could restrict passive membrane diffusion. LTS0212803 with a relatively low molecular weight (264.32 g/mol), TPSA of 66.76 A^2^ and Consensus logP of 1.44 with 4 hydrogen bond acceptors, 2 hydrogen bond donors and 1 rotatable bond, indicated a smaller and less flexible structure that could be conducive to membrane permeability. The radar analysis shows the structural heterogeneity of the predicted leads and suggests that Prenylated Flavanonol (LTS0196517) and LTS0218789 exhibited balanced physicochemical properties and drug-like characteristics comparable to that of positive control Orlistat.

## Molecular dynamic simulation analysis:

To understand the molecular basis of ligand stability within the binding pocket, the interaction profiles of 1LPB-Prenylated Flavanonol (LTS0196517) Complex and 1LPB-Orlistat Complex were analyzed using the MD simulation trajectories. The Prenylated Flavanonol showed a robust and highly persistent interaction network, characterized by strong hydrogen bonds and π-π interactions (Figure 6 - see PDF). The Flavanonol was observed to form strong and long-lasting hydrogen bonds with Ser152 (96%), Asp79 (94%), Phe77 (82%) indicating the ligand to be stable during the course of the simulation. The Flavanonol was observed to maintain interactions with Ser152 and Asp79, crucial residues within the catalytic triad. Further, the aromatic rings of the Flavanonol were observed to form stable π-π interactions with Phe 215 (82%) and Partial interaction with Tyr114 (42%). These forces play an important role in increasing the binding stability by reinforcing hydrophobic packing, restricting ligand rotation and reducing conformational entropy. Orlistat showed intermittent hydrogen bonding with residues Ser152 and Tyr114, in addition to several water mediated interactions that connected the ligand to catalytic site residues. However, the occupancy of these hydrogen bonds was comparatively low, typically fluctuating between 10-18 % throughout the simulation. Hydrophobic interactions were observed with residues Phe77, Phe215, and Leu153 (Figure 6 - see PDF). These interactions facilitate accommodation of the ligands within the hydrophobic pocket of the enzyme the reliance on water-mediated interactions suggests that the ligands may have experienced minor positional adjustments within the binding pocket during the simulation.

## RMSD:

To evaluate the dynamic stability of the 1LPB-Prenylated Flavanonol Complex and 1LPB-Orlistat Complex, RMSD analysis was performed for 200 ns by monitoring both the protein backbone and ligand stability relative to the protein. Results show that the protein backbone remained structurally stable throughout the simulation in both the complexes. The 1LPB-Prenylated Flavanonol complex (Figure 7A - see PDF) exhibited a more stable behavior for the entire period of simulation. The protein backbone RMSD stabilized at 1.62 Å -2.8 Å after a brief equilibration period in the initial 20 ns. Later in the simulation, the protein structure showed a lower RMSD of 3.0 Å, indicating a stable conformation state. A low ligand RMSD during the equilibration phase was observed varying between 0.15 Å and 1.5 Å during the simulation. Results indicate that Prenylated Flavanonol is retained in the binding pocket maintaining stable contacts with residues surrounding the binding pocket.

The RMSD of the 1LPB-Orlistat complex (Figure 7B - see PDF) initially remained constant and was later observed to fluctuate after around 80ns of simulation from about 1.2 Å and 3.5 Å to 3.5 Å - 4.0 Å indicating a moderate conformational change. Further, after 90ns, the RMSD was observed to be unstable probably due to weaker or less stable binding with the active site. The relatively reduced ligand deviations and regulated protein variations in the 1LPB-Prenylated Flavanonol complex indicates more binding stability in comparison to the Orlistat complex.

## RMSF:

To gain further insight into the flexibility of individual amino acid residues and the structural stability of 1LPB-Prenylated Flavanonol and 1LPB-Orlistat Complexes, root mean square fluctuation (RMSF) analysis was performed. In both the complexes, most of the residues were observed to fluctuate in the range of 0.5 Å - 1.5 Å, suggesting that the overall protein structure remained stable during the period of simulation. A distinct peak in residue fluctuations was observed in both the complexes around residues 240-260, highlighting a highly dynamic region of the protein. The 1LPB-Prenylated Flavanonol complex (Figure 8A - see PDF) also displayed increased fluctuations, the magnitude of the fluctuations was slightly lower and the peak appeared more confined. While, in the 1LPB-Orlistat complex, (Figure 8B - see PDF) the RMSF exceeded 5.0 Å, particularly for the side chains, indicating considerable flexibility. Moderate fluctuations were also observed across residues 300-420 in both the complexes. The 1LPB-Orlistat complex showed more dispersed fluctuations across the loop regions, whereas, the 1LPB-Prenylated Flavanonol complex exhibited more localized and controlled movements. The reduced residue-level flexibility observed in this complex suggests stronger and more persistent interactions between the ligand and the protein, indicating that the prenylated flavanonol may provide enhanced stabilization of the pancreatic lipase structure compared to Orlistat.

## Radius of Gyration (Rg):

The radius of gyration (Rg) was measured to assess the structural compactness and conformational stability of the protein -ligand complexes throughout the period of molecular dynamics simulation. The 1LPB-Prenylated Flavanonol complex (Figure 9A - see PDF) showed a significantly narrower distribution, primarily localized between 6.0 Å and 6.4 Å. Following a brief equilibration period of approximately 20 ns, the system reached a stable state, preserving a consistent compactness throughout the simulation. The lack of substantial oscillations indicates enhanced preservation of mass distribution and diminished domain mobility. In the 1LPB-Orlistat complex (Figure 9B - see PDF) the radius of gyration (Rg) values exhibited considerable variation, spanning roughly 4.8 Å - 7.2 Å. The simulation revealed several phases of structural compaction and expansion. A temporary reduction in Rg values between 80-130 ns suggested a temporary tightening of the structures, followed by pronounced expansion phases at around 140-160 ns, during which the Rg values exceeded 7.0 Å. These wider oscillations suggest increased global flexibility and dynamic structural rearrangements. These alternating transitions are indicative of significant conformational stability and domain mobility throughout the simulation.

## Conclusion:

We describe the use of transfer learning with structure-based computational techniques for the identification of potential human pancreatic lipase inhibitors. The transfer learning model developed exhibited a strong predictive capability, achieving a validation performance of R^2^ =0.911 and MAE =0.165, indicating reliable prediction of bioactivity values. Application of this optimized model enabled efficient screening of 109,669 compounds from the LOTUS natural product database, from which 17,309 compounds were predicted to possess inhibitory activity (pIC_50_ >= 6) and selected for further structure-based evaluation. Subsequently Molecular docking showed that prenylated flavanonol displayed the highest binding affinity with an XP docking score of 9.85 kcal/mol, which was considerably stronger than that of the reference inhibitor orlistat (3.81 kcal/mol). Moreover, 200ns molecular dynamics simulations confirmed that prenylated flavanonol forms a stable within the catalytic site of pancreatic lipase, highlighting its potential as a promising natural lead compound for anti-obesity drug development.

## Disclosure statement:

No potential conflict of interest was reported by the authors.

## CRediT authorship contribution statement:

C. Tamilselvan: Collection of data, Data curation, Methodology, Validation, Visualization, Interpretation of data, Writing - Original draft, review, editing. B. S. Lakshmi: Conceptualization

## Figures and Tables

**Table 1:** Binding energy and H-bond interactions of compounds with human pancreatic lipase using Molecular Docking

**Compound ID**	**Compound Name**	**XP Score (kcal/mol)**	**Interacting Residues**
LTS0196517	Prenylated Flavanonol	-9.85	H-bond: THR115, SER152; π-π: PHE77
LTS0218789	5,6-dihydroxy-2-(4-hydroxyphenyl)-7[(2S,3R,4R,5R,6S)-3,4,5-trihydroxy-6methyloxan-2-yl]oxychromen-4-one	-8.5	H-bond: ASP79, TYR114
LTS0199947	(1R,2R,3S,7R,11S)-11-hydroperoxy2-hydroxy-1-methyl-6-methylidene-4oxatricyclo[8.3.1.03,7]tetradec-9-en-5-one	-7.64	H-bond: PHE77, SER152; π-π: HIS263
LTS0212803	(1R,8S,11S)-1,11-dihydroxy-11-isopropyl-8-methyl-2-oxatricyclo[6.3.1.04,12]dodeca-4(12),6-dien-5-one	-6.8	H-bond: PHE77
CID3034010	Orlistat (Control)	-3.81	H-bond: PHE77, SER152, LEU153
